# A system of personality-oriented treatment of stress-related disorders in war-affected adults

**DOI:** 10.1192/j.eurpsy.2025.1958

**Published:** 2025-08-26

**Authors:** H. Kozhyna, O. Druz, I. Chernenko, K. Zelenska, K. Marushchenko, A. Kondratenko

**Affiliations:** 1 Department of Psychiatry, Narcology, Medical Psychology and Social Work, Kharkiv National Medical University, Kharkiv; 2 Psychiatric Clinic (with rooms for drug-addicted), National Military Medical Clinical Centre “Main Military Clinical Hospital” of the Ministry of Defense of Ukraine; 3 Department of Military Therapy, Ukrainian Military Medical Academy, Kyiv, Ukraine

## Abstract

**Introduction:**

War is the most powerful psychosocial stressor affecting all segments of modern Ukrainian society.

**Objectives:**

It was conducted a comprehensive examination of 176 patients of both sexes: Group I consisted of 101 military personnel; Group II - 97 volunteers; Group III - 95 IDPs in order to develop a system of personality-oriented treatment of stress-related disorders in persons affected by war.

**Methods:**

Clinical and psychopathological examination, which included a structured interview and patient’s observation aimed at studying influence of socio-psychological and biological factors on development of post-stress disorders. Psychodiagnostic method include use: M-PTSD; HADS; HAM-A, HAM-D; Questionnaire of neuropsychological stress by T.A. Nemchin; State-Trait Anxiety Invertory; Methods of diagnosing coping behaviour in stressful situations; Impact of Event Scale-Revised; Clinical Administered PTSD Scale-CAPS; Traumatic Stress Questionnaire; Colombian Suicide Intentions Severity Scale; Methods for determining suicide risk and assessing self-awareness of death in patients with depressive disorder (Kozhyna H.M., Zelenska K.O., 2015); Methods for ‘Diagnosing the level of social frustration (Wasserman L.I., modified by Boyko V.V., 2002).

**Results:**

Clinical structure of stress-related disorders was presented by PTSD and adjustment disorders. Clinical structure of PTSD was represented by anxious, dysphoric, asthenic and somatoform syndromes. System of personality-oriented treatment was developed, including differentiated use of psychopharmacotherapy, psychotherapy and psychoeducation; creation of re-adaptive atmosphere; formation of health-centered lifestyle and based on a salutogenic approach. Pharmacotherapeutic component of developed program included differentiated, targeted use of SSRIs, SNRIs, antipsychotics, tranquilizers and anxiolytics. Psychotherapeutic program was based on identification of dominant resource channels for overcoming stress and finding inner stability using integrative model of psychological survival after severe stress, Basic Ph. Psychotherapeutic support included trauma-focused CBT for all patients, EMDR therapy with additional use of Pucelik Consulting Group’s PTSD Self-Management Program for servicemen patients in Group I; individual crisis therapy for patients in Group II; and interpersonal therapy for patients in Group III. For anxious depressive reactions, CBT and art therapy were used for all patients; with the additional use of problem-solving therapy for patients of Group I; Group II - individual crisis therapy; Group III - mindfulness techniques, relaxation training.

**Conclusions:**

Effectiveness of developed system of personality-oriented treatment of stress-related disorders was proved, and positive dynamics of mental state, reduction of psychopathological symptoms, increased resistance, reduced levels of social and psychological frustration were established.

**Disclosure of Interest:**

None Declared

